# Supplementary material for: Identification of photoperiod-induced specific miRNAs in the adrenal glands of Sunite sheep (Ovis aries)
Source: Front Vet Sci. 2022 Jul 22;9:888207. doi: 10.3389/fvets.2022.888207 (PMC9354845; doi:10.3389/fvets.2022.888207)
Supplement: Supplementary file 1 [file Data_Sheet_1.ZIP › Supplementary file/miRDeep2 file.zip!/miRDeep2 file/Know_Structure/SP42A3/oar-mir-412.pdf]

miRBase precursor : oar-mir-412
Total read count : 308
oar-miR-412-3p read count : 302
oar-miR-412-5p read count : 4
remaining reads : 2

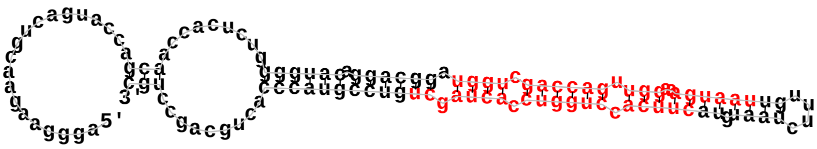

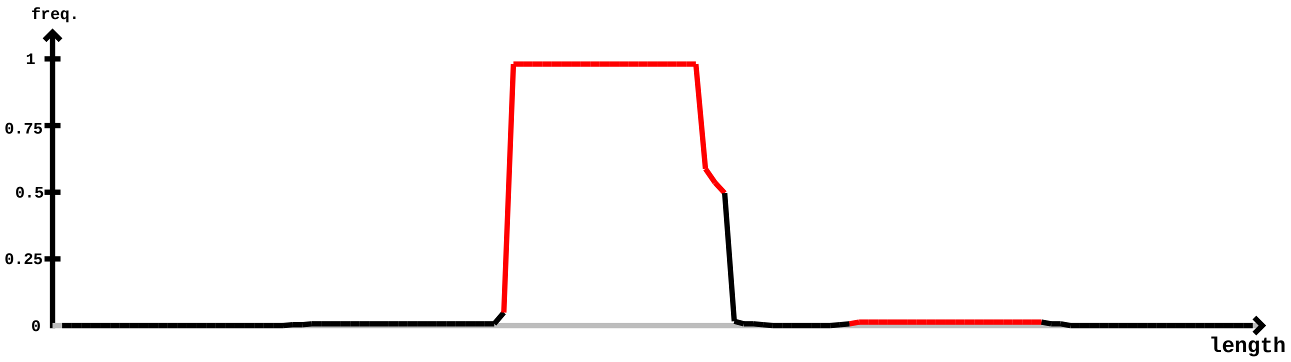


```
                                                                                     oar-miR-412-5p
                                              oar-miR-412-3p
5'- agggaagaacgucaguaccagcaaccacucugggguacaggacggauggucgaccaguuggaaaguaauuguuucuaauguacuucaccugguccacuagcuguccguacccacugcagccugc  -3'   exp
    ..........(.((((..(((........)))((((((.((((((.((((.((((((.(((..((((((((....)))).))))))).))))))).)))).))))))))))))))))).......   reads   mm
    .......................accacucugggguacaggacgga..............................................................................   1       0
    ........................cacucugggguacaggacgga..............................................................................   1       0
    .............................................auggucgaccaguuggaaagu..........................................................   12      0
    .............................................auggucgaccaguuggaaagua.........................................................   1       0
    ..............................................uggucgaccaguuggaaagu..........................................................   109     0
    ..............................................uggucgaccaguuggaaagua.........................................................   15      0
    ..............................................uggucgaccaguuggaaaguaa........................................................   12      0
    ..............................................uggucgaccaguuggaaaguaau.......................................................   148     0
    ..............................................uggucgaccaguuggaaaguaauu......................................................   3       0
    ..............................................uggucgaccaguuggaaaguaauugu....................................................   1       0
    ..............................................uggucgaccaguuggaaaguaauuguu...................................................   1       0
    ................................................................................uacuucaccugguccacuagcu......................   1       0
    .................................................................................acuucaccugguccacuagcu......................   1       0
    ..................................................................................cuucaccugguccacuagcugu....................   2       0
```
